# hiPSC hepatocyte model demonstrates the role of unfolded protein response and inflammatory networks in α_1_-antitrypsin deficiency

**DOI:** 10.1016/j.jhep.2018.05.028

**Published:** 2018-10

**Authors:** Charis-Patricia Segeritz, Sheikh Tamir Rashid, Miguel Cardoso de Brito, Maria Paola Serra, Adriana Ordonez, Carola Maria Morell, Joseph E. Kaserman, Pedro Madrigal, Nicholas R.F. Hannan, Laurent Gatto, Lu Tan, Andrew A. Wilson, Kathryn Lilley, Stefan J. Marciniak, Bibek Gooptu, David A. Lomas, Ludovic Vallier

**Affiliations:** 1 Wellcome Trust and MRC Cambridge Stem Cell Institute, Department of Surgery, University of Cambridge, UK; 2 Cambridge Institute for Medical Research, University of Cambridge, UK; 3 Centre for Stem Cells and Regenerative Medicine & Institute for Liver Studies, King’s College London, UK; 4 UCL Respiratory, University College London, UK; 5 Center for Regenerative Medicine (CReM) of Boston University and Boston Medical Center, Boston, MA 02118, USA; 6 Cambridge Centre for Proteomics, Department of Biochemistry, University of Cambridge, Building O, Downing Site, Cambridge CB2 1QW, UK; 7 Wellcome Trust Sanger Institute, Genome Campus Hinxton, UK; 8 NIHR Leicester BRC-Respiratory and Leicester Institute of Structural & Chemical Biology, University of Leicester, UK; 9 ISMB/Birkbeck & UCL, University of London, UK; 10 Division of Asthma, Allergy and Lung Biology, King’s College London, UK

**Keywords:** Hepatocyte, Inherited liver disease, Human-induced pluripotent stem cell, α_1_-Antitrypsin deficiency, Inflammation

## Abstract

**Background & Aims:**

α_1_-Antitrypsin deficiency (A1ATD) is an autosomal recessive disorder caused by mutations in the *SERPINA1* gene. Individuals with the Z variant (Gly342Lys) retain polymerised protein in the endoplasmic reticulum (ER) of their hepatocytes, predisposing them to liver disease. The concomitant lack of circulating A1AT also causes lung emphysema. Greater insight into the mechanisms that link protein misfolding to liver injury will facilitate the design of novel therapies.

**Methods:**

Human-induced pluripotent stem cell (hiPSC)-derived hepatocytes provide a novel approach to interrogate the molecular mechanisms of A1ATD because of their patient-specific genetic architecture and reflection of human physiology. To that end, we utilised patient-specific hiPSC hepatocyte-like cells (ZZ-HLCs) derived from an A1ATD (ZZ) patient, which faithfully recapitulated key aspects of the disease at the molecular and cellular level. Subsequent functional and “omics” comparisons of these cells with their genetically corrected isogenic-line (RR-HLCs) and primary hepatocytes/human tissue enabled identification of new molecular markers and disease signatures.

**Results:**

Our studies showed that abnormal A1AT polymer processing (immobilised ER components, reduced luminal protein mobility and disrupted ER cisternae) occurred heterogeneously within hepatocyte populations and was associated with disrupted mitochondrial structure, presence of the oncogenic protein AKR1B10 and two upregulated molecular clusters centred on members of inflammatory (IL-18 and Caspase-4) and unfolded protein response (Calnexin and Calreticulin) pathways. These results were validated in a second patient-specific hiPSC line.

**Conclusions:**

Our data identified novel pathways that potentially link the expression of Z A1AT polymers to liver disease. These findings could help pave the way towards identification of new therapeutic targets for the treatment of A1ATD.

**Lay summary:**

This study compared the gene expression and protein profiles of healthy liver cells and those affected by the inherited disease α_1_-antitrypsin deficiency. This approach identified specific factors primarily present in diseased samples which could provide new targets for drug development. This study also demonstrates the interest of using hepatic cells generated from human-induced pluripotent stem cells to model liver disease *in vitro* for uncovering new mechanisms with clinical relevance.

## Highlights

• Modelling liver disease due to α_1_-antitrypsin deficiency reveals disruption of the ER into isolated cisternae.
• Comparative ‘omics’ between diseased hiPSC-derived and wild-type hepatocytes provides insights into disease mechanisms.
• AKR1B10 was identified as a putative biomarker for α_1_-antitrypsin deficiency.

## Graphical abstract

**Figure:**
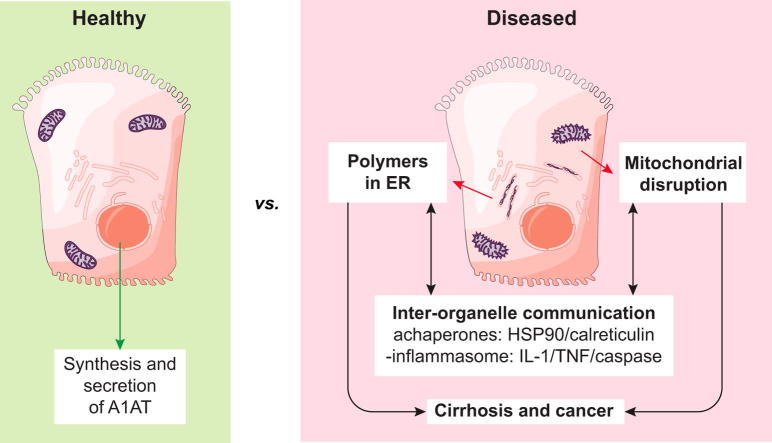

## Introduction

α_1_-Antitrypsin (A1AT) is a 52 kDa protein encoded by the *SERPINA1* gene synthesised primarily by hepatocytes.^1^ Secreted into the blood stream, it acts to control the function of neutrophil elastase, particularly in the lung.^2^ A1AT also exerts anti-apoptotic and anti-inflammatory properties during inflammation and hepatic injury. Most people carry the wild-type M allele, while the rarer Z variant (found in 1–3% of the population), is associated with the most common and severe form of clinically significant A1AT deficiency (A1ATD).^3^ The Z allele is caused by a Glu342Lys mutation in exon 5 of the *SERPINA1* gene, leading to conformational instability within the protein.^4^ Approximately 70% of synthesised Z A1AT is degraded by intracellular quality control mechanisms, 15% is secreted whilst the remaining 15% accumulates in hepatocytes as ordered polymers. These polymers are associated with neonatal hepatitis, cirrhosis and hepatocellular carcinoma. Furthermore, the significant reduction in circulating plasma A1AT levels leads to uncontrolled proteolytic activity within the lung and development of early onset panlobular emphysema.^5^

Despite the recognition of A1ATD over 50 years ago,^6^ the detailed molecular mechanisms linking A1AT polymer accumulation to the development of liver disease remain poorly understood. This has been hampered by the availability of primary human hepatocytes expressing wild-type and mutant forms of A1AT capable of surviving in culture long-term. Although the use of animal models and artificial A1AT-expressing cell systems have contributed significantly to improved understanding of the disease processes,[7], [8], [9] multiple gene copies, interference of endogenous animal antiproteases, lack of pathological features post-polymer accumulation, and the absence of endogenous promoters to activate gene expression represent challenges for translating new findings in these model systems to man.

The advent of human-induced pluripotent stem cells (hiPSCs)^10^ has provided an exciting platform to address these obstacles. Indeed, PiZZ hiPSCs (ZZ-hiPSCs) derived from patients with A1ATD were shown to differentiate into hepatocyte-like cells (ZZ-HLCs) that recapitulate the modified post-translational processing and secretion kinetics of mutant A1AT.[11], [12] Furthermore, patient-specific HLCs were genetically corrected to erase the disease signature (RR-HLCs)^13^ and so generated a perfect control cell line with which to perform mechanistic studies.

Using these tools, we first established the suitability of HLCs to study the mechanisms of A1ATD by benchmarking their gene expression, protein synthesis and metabolic activity against primary hepatocytes. Then, by comparing the transcriptome and endoplasmic reticulum (ER)-enriched proteome of ZZ- and RR-HLCs, we validated the platform’s efficiency for identifying novel biomarkers and pathways involved in mediating the disease processes that may be of immediate clinical relevance.

## Materials and methods

### hiPSC culture and hepatic differentiation

All hiPSC lines used in this study have were derived as previously described^11^ under Addenbrooke’s Hospital Ethics reference number 08/H0311/201; R&D No. A091485. These cells were maintained at 37 °C in humidified incubators supplemented with 5% v/v carbon dioxide and were differentiated using our established hepatic differentiation protocol with minor modifications:^14^ Cells were differentiated at 5% oxygen and two splitting steps allowed for scale-up and prolonged maturation of differentiating cells (Fig. S1A). During the splitting steps on day 8 and day 25, cells were washed with PBS and dissociated with TrypLE™ (Life Technologies, 12563–029) for 20–45 min at 37 **°**C. Harvested cells were centrifuged at 100 × g for 3 min. Cells were resuspended in RPMI (day 8 split) or HepatoZYME-SFM (day 25) (Thermo Fisher, 17705–021). Cells were counted and resuspended in RPMI and Activin (day 8 split) or HepatoZYME-SFM (Thermo Fisher, 17705–021), OSM and HGF (day 25 split), supplemented in each case with ROCK inhibitor (1 μL/ml) to a concentration of 210,500 cells/cm^2^.

### Primary hepatocyte procurement

Commercially plated human primary hepatocytes from two donors were purchased from Biopredic International (France). The cells exhibited viabilities between 88–93% after isolation, fulfilled the manufacturer’s requirements of Phase I and II-dependent enzyme activities and were cultured for 48 h in hepatocyte medium prior to harvesting. Fresh human primary hepatocytes were kindly provided by Dr. Roque Bort with approval of the hospital’s ethics committee (Instituto de Investigación Sanitaria La Fe, Valencia, Spain). Upon isolation, hepatocytes were snap frozen and the resulting cell pellets were directly lysed for RNA extraction.

### RNA-seq

Library preparation of RNA samples was performed with the Illumina TruSeq RNA Sample Preparation V2 assay. Samples were multiplexed on two lanes with 40 bp (HLCs) or 75 bp (PiZZ/PiMM) read lengths, single strand and single-end (HLCs) or paired-end (PiZZ/PiMM) reads and generated ∼16.5 million reads per sample on the Illumina HiSeq 2000. Bioinformatic analysis were carried out following standard procedures^11^ and of raw data are available on arrayexpress accession number E-MTAB-6781.

## Results

### hiPSC-derived hepatocyte-like cells are suitable for modelling human disease

**Fig. 1:**
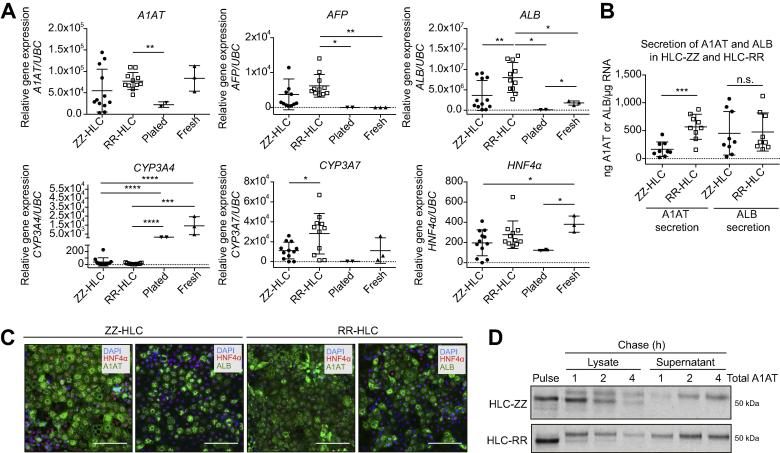
**Validating a cellular platform suitable for in-depth disease characterisation.** (A) qPCR analyses for the denoted genes in ZZ- (mutant, n = 12) and RR- (corrected, n = 12) HLCs *vs.* positive controls (plated and fresh, cryopreserved human primary hepatocytes, n = 2–3). (B) DELFIA of secreted α_1_-antitrypsin and albumin in ZZ- and RR-HLCs; n = 3–9. (C) Immunostaining of ZZ and RR-HLCs for HNFα, α_1_-antitrypsin or albumin. Scale bar: 100 μm. (D) Pulse-chase analysis: Radioactively labelled, newly-synthesised protein was tracked from the intracellular (lysate) to extracellular space (supernatant) at 1 h, 2 h and 4 h. HLCs, hepatocyte-like cells; qPCR, quantitative PCR. Statistical analyses of (A) and (B) by unpaired, parametric *t* test: n.s. (non-significant), *p <0.05, **p ≤0.01, ***p <0.001, ****p ≤0.0001.

We have previously generated disease specific hiPSCs (ZZ-hiPSCs)^11^ and created an isogenic wild-type line (RR-hiPSC) using genome editing.^13^ Once differentiated, ZZ-derived hepatocyte-like cells (ZZ-HLC) displayed intracellular polymer retention whilst corrected cells (RR-HLC) secreted normal levels of A1AT. Thus, RR-HLC represent the ideal control for molecular studies, since phenotypic differences observed between both cell types should exclusively be attributable to the misfolding of mutant A1AT in HLC-ZZs and not to variability between hiPSCs lines of different genetic backgrounds.^15^ To further validate this hypothesis, we compared the hepatic function of ZZ-HLCs and RR-HLCs using primary hepatocytes freshly isolated, cryopreserved or freshly plated as a positive control. Of note, we applied an optimised protocol^14^ allowing HLCs to differentiate for an extended period of time, increasing their functional repertoire (Fig. S1A-C). As expected, HLCs expressed a diversity of hepatic markers including *A1AT*, *ALB* and *HNF4α* at levels equivalent to primary hepatocytes (Fig. 1A-C). They also displayed *CYP3A4* expression and activity although at significantly lower levels than primary cells (Fig. 1A, Fig. S1B). Conversely, HLCs showed expression of foetal markers *CYP3A7* and *AFP* at higher levels (Fig. 1A) thereby confirming their foetal identity.^16^ More importantly, ZZ-HLCs and RR-HLCs expressed comparable levels of hepatic markers (Fig. 1A). These observations were confirmed by immunostaining and DELFIA®, showing these markers to be expressed at equivalent levels in both cell types with the exception of A1AT, which was synthesised in lower concentrations into ZZ-HLCs culture media (Fig. 1B-C). To confirm this observation, A1AT trafficking in ZZ-HLCs was assessed by growing HLCs in the presence of radioactively labelled [^35^S] Cys/Met and by subsequently tracking its transition from the intracellular to the extracellular space over the course of 4 h. The band intensities of these pulse-chase experiments revealed a slower rate of glycoprotein maturation (*e.g.* sialic acid residue incorporation) and trafficking of A1AT to the extracellular space in ZZ-HLCs (Fig. 1D). Whilst wild-type A1AT was almost fully secreted after 4 h in RR-HLCs, mutant A1AT in ZZ-HLCs was mostly degraded or retained intracellularly during the same time period.^17^ Taken together, these results demonstrate that ZZ-HLCs and RR-HLCs have reproducible biological characteristics *in vitro,* except for the processing of A1AT, and therefore provide a suitable control-study pair for in-depth characterisation of the disease pathophysiology of A1ATD.

### ZZ-HLCs abnormally process A1AT polymer in a heterogenous, ER-dependent manner

**Fig. 2:**
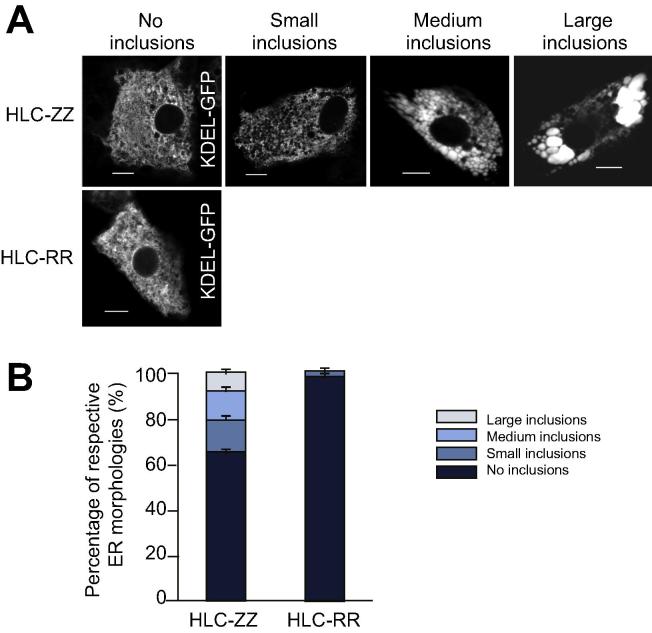
**ZZ-HLCs display heterogenous abnormal ER morphology.** (A) ER marker GFP-KDEL shows the different types of abnormal ER morphologies observed in ZZ-HLCs *vs.* RR-HLCs. (B) Quantification of each ER morphology. ER, endoplasmic reticulum; GFP, green fluorescent protein; HLCs, hepatocyte-like cells.

**Fig. 3:**
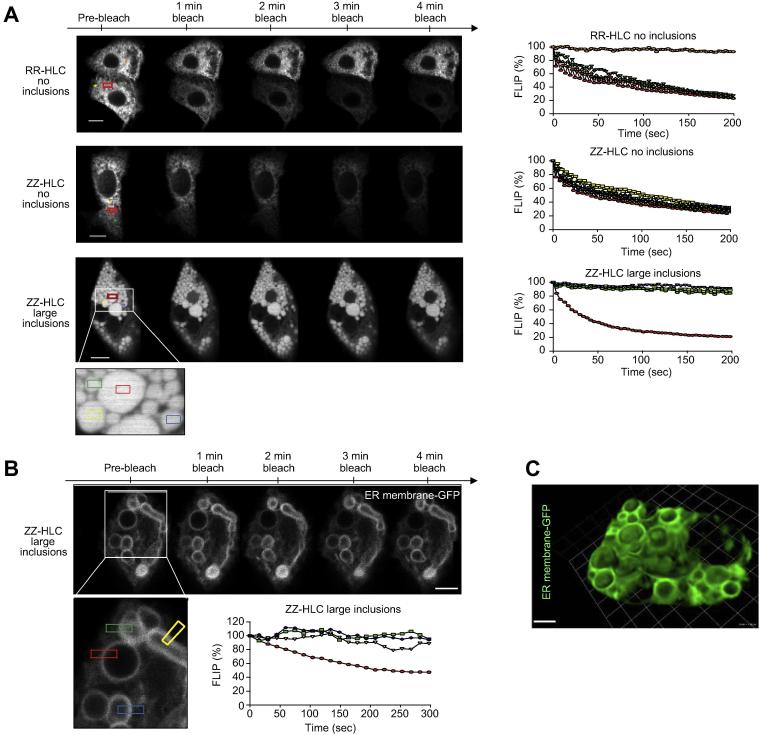
**Fluorescence loss in photobleaching shows absence of intracellular exchanges between ER inclusions.** (A) FLIP of ZZ- and RR-HLCs transfected with KDEL-GFP: Pre-bleach fluorescence was determined before bleaching. Fluorescence was monitored within equidistant areas (stars) from the area of bleach (red box). Graphs show normalised FLIP plots with pre-bleach intensities set to 100%. n = 5. (B) FLIP of ZZ-HLCs with large inclusions transfected with CytERM. Graph shows normalised FLIP plot with pre-bleach intensities set to 100%. n = 3. (C) 100 μm Z-tack image of ZZ-HLCs. Scale bars: 10 μm. FLIP, fluorescence loss in photobleaching; GFP, green fluorescent protein; HLCs, hepatocyte-like cells.

**Fig. 4:**
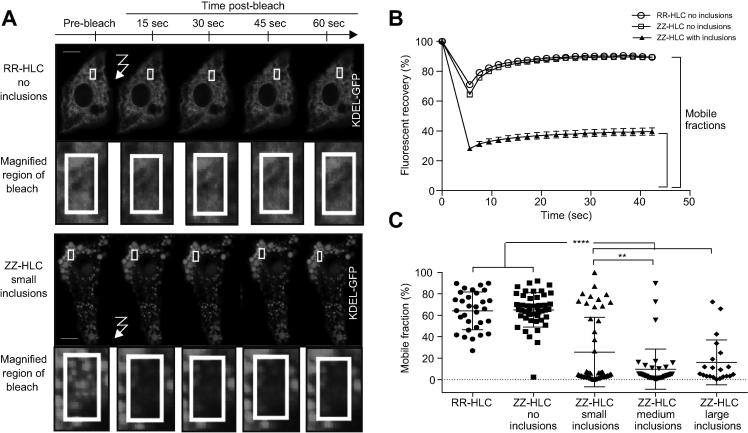
**Fluorescent recovery after photobleaching confirming decreased ER protein mobility in ZZ-HLCs.** (A) KDEL-GFP transfected cells were exposed to a single bleaching event (white box). (B) Recovery of GFP fluorescence by way of diffusion into the bleached area was measured by tracking fluorescence within the bleached area. (C) Slope of the fluorescent recovery curve and the mobile fraction indicate a significantly lower degree of protein mobility and higher viscosity in ZZ-HLCs with inclusions. Statistical analysis by unpaired, parametric *t* test: **p ≤0.01, ****p ≤0.0001. ER, endoplasmic reticulum; FRAP, fluorescent recovery after photobleaching; GFP, green fluorescent protein; HLCs, hepatocyte-like cells.

To investigate the impact of A1AT protein accumulation on cellular trafficking, we studied the hepatic ER – the site at which mutant A1AT proteins aggregate – at a single cell level. ZZ- and RR-HLCs were transfected with the ER marker, green fluorescence protein (GFP)-KDEL, that has been shown to co-localise with A1AT polymers.^9^ Transfections of HLCs with the GFP-KDEL construct yielded a population of RR-HLCs which all contained normal, reticular ER morphologies (Fig. 2A). In contrast, ZZ-HLCs frequently displayed large ER inclusions. We classified these as inclusions of small, medium or large size and quantified the percentage of cells containing each sub-type (Fig. 2B). Overall, 40% of ZZ-HLCs showed inclusions, whilst the remaining 60% of cells exhibited a normal ER. We next analysed the effect of intracellular inclusions on ER architecture using “fluorescence loss in photobleaching” (FLIP) and “fluorescence recovery after photobleaching” (FRAP) to track live, GFP-KDEL transfected ZZ-HLCs and RR-HLCs.^18^ This allowed an evaluation of the cell’s ER connectivity (FLIP) and luminal protein mobility (FRAP) by monitoring the diffusion rates of inert fluorescent probes throughout different ER morphologies. Repeated photobleaching of a pre-defined fluorescent region resulted in complete depletion of fluorescence in inclusion-free ZZ- and RR-HLCs, while neighbouring cells were unaffected (Fig. 3A). Of particular note was the small, consistent recovery of fluorescence in between the bleaching events, suggesting rapid diffusion of unbleached KDEL-fluorophores back to the area of interest. These observations reinforced our previous results obtained on transformed cell lines^19^ by showing that in wild-type HLCs, ER proteins can sample distant compartments of the ER by diffusion when enclosed by connected, tubular ER luminae. In contrast, in inclusion-bearing ZZ-HLCs, FLIP within large inclusions resulted in the localised loss of KDEL-GFP fluorescence with no recovery of fluorescence after bleaching events. Furthermore, neighbouring inclusions were not affected, indicating walled-off ER cisternae with no or restricted ability for protein diffusion or interactions between them. These observations were further confirmed using another fluorescent protein chimera, the cytoplasmic domain of an ER signal anchor membrane protein (CytERM).^20^ FLIP performed on ZZ-HLCs with large inclusions transfected with fluorescent CytERM resulted in complete loss of fluorescence of the inclusion’s ER membrane, whilst neighbouring inclusions were unaffected (Fig. 3B). Finally, fragmentation and isolation of ER inclusions were confirmed using Z-stack images captured across ZZ-HLCs transfected with the fluorescent ER membrane protein (Fig. 3C). The mobility of ER proteins was then quantified using FRAP assays.^18^ The pre-bleach fluorescence level of a selected region of interest inside the cytoplasm was normalised to 100% and a single bleaching event was performed within the region of interest. Diffusion rates of unbleached KDEL-GFP molecules from adjacent areas into the bleached region were determined by monitoring the restoration of fluorescent levels into the region of interest. We observed that the patterns of fluorescent recovery were similar within regions of reticular ER in RR- and ZZ-HLCs (Fig. 4A). Mapping the percentage of fluorescent rescue to the area of bleaching showed almost immediate recovery at approximately 60%, which was further restored to nearly 90% in RR-HLCs. In contrast, inclusion-bearing ZZ-HLCs showed recovery of only 30–40% of the original KDEL-GFP fluorescence. Regression analysis to reveal the rates of recovery, *i.e.* the slope of the respective fluorescent recovery curves, confirmed faster diffusion rates of proteins in areas of connected reticular ER compared to the rates seen in areas with disrupted ER morphologies (RR-HLC: 2.92 ± 0.75, ZZ-HLC no inclusions: 3.99 ± 1.03, ZZ-HLC with inclusions 1.23 ± 0.22). There appeared to be no or very little recovery of fluorescence in bleaching of larger inclusions, suggesting a size-dependent isolation of inclusions (Fig. 4B). To quantify the percentage of total fluorescent molecules that contribute to the fluorescent recovery of the photobleached area during the assayed time frame, we assessed the mobile fractions associated with individual ER morphologies. These showed that protein viscosities of reticular ER morphologies were significantly lower than that of fractionated ER sites (*p* ≤0.0001) (Fig. 4C). In summary, these data showed that polymer accumulation in A1ATD reduced ER protein diffusion rates suggestive of immobilised ER components, reduced protein mobilities and/or disrupted ER cisternae in inclusion-bearing ZZ-HLCs.^21^

### Transcriptome and proteome analyses suggest ZZ-HLCs acquire mitochondrial abnormalities and are poised for malignancy

**Fig. 5:**
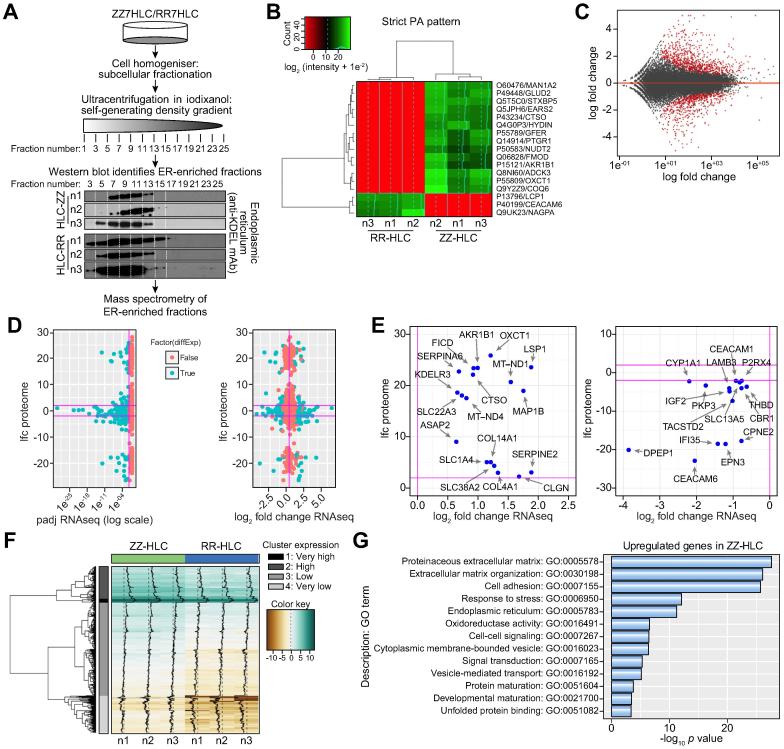
**Proteomic and RNA-seq analyses capture novel disease signatures.** (A) Experimental process for proteomic analyses. (B) List of proteins identified in the ER proteome (green: present) and (red: absent) between ZZ- and RR-HLC. (C) Microarray plot shows log_2_ fold changes in gene expression between ZZ- and RR-HLCs over the mean of normalised counts. Significant genes with Benjamini-Hochberg FDR < 0.05 are shown in red. (D) 319 gene-protein pairs with differential abundances and gene expression were identified (blue, adjusted *p* values >0.05 in red). (E) Most differentially expressed gene/protein pairs upregulated (top) or downregulated (bottom). Horizontal lines: parameter boundaries of log2-fold change thresholds >2 and <−2. (F) Data clustering showed the FPKM-based genes upregulated in ZZ-HLCs mapped into four groups: very high, high, low or very low expression. (G) Enriched gene ontology terms (*p* value <0.05) obtained from BioMart for the analysis of genes upregulated in ZZ-HLCs. ER, endoplasmic reticulum; FDR, false discovery rate; FPKM, fragments per kilobase of transcript per million mapped reads; HLCs, hepatocyte-like cells.

Given the abnormalities in protein mobility observed in the ER, we hypothesised that polymer accumulation in ER inclusions could be associated with specific proteins inhibiting A1AT polymer degradation and eliciting stress responses.[22], [23], [24] To investigate this hypothesis, we performed proteomic analyses on ER fractions isolated from ZZ- and RR-HLCs (Fig. 5A). Three independent batches of ZZ- and RR-HLCs were fractionated into their subcellular ER fractions and analysed by label-free mass spectrometry. We identified 2,683 unique proteins in the ZZ- and RR-HLC ER proteomes, which were enriched most significantly for the ER (adjusted *p* value: 7.1–91) and mitochondria (adjusted *p* value: 1.6–172). Among these proteins, 14 were exclusively identified in all three batches of ZZ-HLCs whilst three proteins were present only in the RR-HLCs (Fig. 5B). By gene ontology (GO) enrichment analysis, these 17 proteins with strict distribution patterns displayed a diversity of functions involved in intracellular trafficking, extracellular matrix organisation and enzymatic activity. Five of these proteins (29%) were attributed to mitochondrial localisation and oxidoreductase activity: GLUD2, GFER, OXCT1, EARS2 and ADCK3 (COQ8A). In order to further understand the implications of A1AT protein accumulation on a whole-cell scale, genome-wide RNA sequencing (RNA-seq) was performed on ZZ-HLCs and RR-HLCs. Statistical analysis revealed 1,675 genes with significantly different expression (false-discovery rate (FDR) <5%), the majority of which were protein-coding genes (93.4%) (Fig. 5C). Of the 1,675 differentially expressed genes, 959 were upregulated in ZZ-HLCs (958 of them with a fold change >1.5), while 716 genes were downregulated in ZZ-HLCs (715 of them with a fold change <−1.5). A total of 319 genes with differential transcript expression were also detected in the mass spectrometry dataset (Fig. 5D). We then combined both proteomic and transcriptomic data sets by considering differential abundances of proteins and genes with log-fold changes >2 or <−2. Candidate markers that fulfilled these parameters included 18 upregulated and 15 downregulated genes/proteins (Fig. 5E), of which 15% were ER-localised proteins (CLGN, KDELR3, COL4A1, COL14A1, CYP1A1). Of note, the low correlation of data points seen under these conditions may be explained by the comparison of whole-cell transcriptomes with subcellular ER-specific proteomes. We next focused on examining the absolute gene expression of the assayed genes and considering ZZ-HLCs as the reference. We found that 20 of the 21 most highly-expressed and differentially upregulated genes in ZZ-HLCs were of mitochondrial origin (Fig. 5F). The majority of their functional repertoire was relevant to oxidoreductase activity and mitochondrial ATP synthesis in the electron transport chain and included NADH dehydrogenases, ATP synthases, cytochromes b and c. The only non-mitochondrial gene in the very highly-expressed, upregulated gene cluster was fibrinogen gamma chain (*FGG*), upregulation of which has previously been associated with hepatic inflammation^25^ and hepatocellular carcinoma.^26^ Other molecules with reported oncogenic associations were also found in the highly-expressed, upregulated cluster and included *H19*,^27^ *SPARC*^28^ and *SERPINE1*.^29^ Furthermore, GO over-representation analyses on all highly-expressed, upregulated genes revealed that the five most enriched and significant GO terms were tied to extracellular matrix genes associated with fibrosis and cirrhosis (*p* values <0.0001), a hallmark of liver disease in A1ATD (Fig. 5G). This included collagen-related genes (*e.g. COL1A1*, *COL1A2*, *COL3A1*, *COL4A1* and *COL5A1*) and genes whose products are involved in the assembly and remodelling of the extracellular matrix such as ADAM metallopeptidases (*e.g. ADAM19*), fibrillin (*e.g. FBN1*), fibronectin (*e.g. FN1*), laminins (*e.g. LAMB1*), thrombospondin (*e.g. THBS1*) and matrix-associated proteins encoded by *SPARC* and *WNT5A*. Taken together, these analyses uncovered highly relevant proteins involved in the response to *SERPINA1* mutations.

### Validation of discoveries from the hiPSC-derived hepatocyte platform

**Fig. 6:**
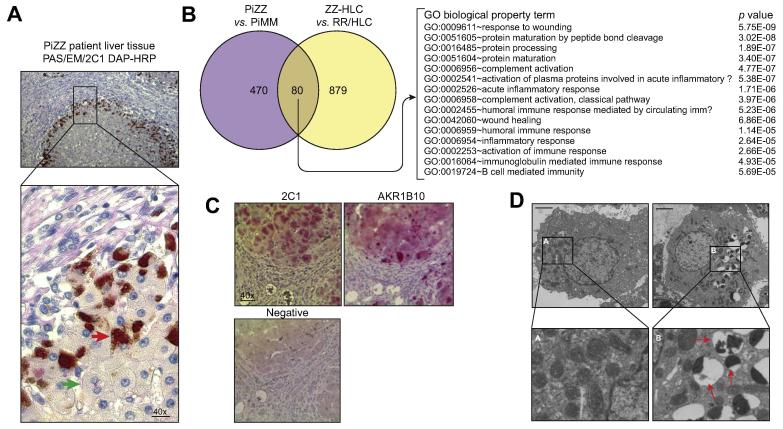
**Clinical relevance of novel disease signatures.** (A) Immunostaining of PiZZ patient liver tissue sections confirmed (green arrow) and absence (red arrow) of PAS-positive α_1_-antitrypsin globules (counter stained with polymer specific 2C1 antibody). Scale bar: 10 μm. (B) 1,675 differentially regulated genes identified by RNA-seq of ZZ *vs.* RR-HLCs were compared to the top 1,675 genes with the lowest *p* values obtained by RNA-seq of one PiZZ *vs.* PiMM primary tissue sample. 80 genes were shared between the two gene lists. (C) AKR1B10 immunostaining of liver sections (n = 3 patients) with clinical backgrounds matching those of the original hIPSC donor. Staining with the 2C1 antibody confirmed presence of polymer in same cells as AKR1B10; negative controls (secondary antibody alone). Scale bar: 20 μm. (D) Electron microscopy comparing mitochondria in ZZ *vs.* RR-HLCs. Organelles with highly compressed morphology yielding heterogeneous electron densities are indicated (red arrows). Scale bar: 2 μm. hiPSC, human-induced pluripotent stem cells; HLCs, hepatocyte-like cells.

We concluded that overall, several new areas of A1AT-deficient biology in HLCs were identified and warranted follow-up. The most significant of which were: i) A1AT polymers accumulated in a heterogenous manner within ZZ-HLC hepatocyte populations, ii) specific proteins associated with predisposition to malignancy were found to be highly upregulated in ZZ-HLCs, iii) mitochondrial biology in ZZ-HLCs was abnormally affected, and iv) ZZ-HLCs displayed activation of important inflammatory and unfolded protein response pathways. To validate the relevance of these findings, we next assessed the accumulation of A1AT polymers in liver tissue from individuals with PiZZ A1ATD^30^ (Fig. 6A). While the majority of hepatocytes exhibited no polymer accumulation, those displaying characteristic A1AT inclusions were mainly located at the periphery of hepatic lobules, thereby validating the cell-to-cell heterogeneity of polymer formation observed in ZZ-HLCs (Fig. 2A). We next compared genes differentially expressed in HLCs (ZZ-HLCs *vs.* RR-HLCs) and primary hepatocytes (PiZZ *vs.* PiMM) and found 80 upregulated genes common to both datasets (Fig. 6B). One of these 80 genes (*AKR1B10*) had a related enzyme product (AKR1B1) present exclusively in the ER-enriched proteome dataset of ZZ-HLCs (Fig. 5B) and a log_2_-fold change of 3.6 and 3.8 in differential gene expression between ZZ/RR-HLCs and PiZZ/PiMM hepatocytes respectively. The clinical significance of this molecule was assessed by immunostaining paraffin-embedded liver sections from three patients with PiZZ whose clinical backgrounds (age, sex and severity of disease) matched those of the original hiPSC line donor. Samples stained positively for AKR1B10 in clusters of cells that were also positive for polymers of A1AT (Fig. 6C). Having demonstrated the potential clinical relevance of the data generated from our *in vitro* model for biomarker discovery, we next sought to explore physiological pathways that may underpin A1AT pathogenesis by focusing on GO terms associated with unfolded protein binding. Utilising STRING v9.1, we surveyed known and predicted protein-protein interactions encoded by those genes upregulated with the highest confidence view (score = 0.900) in ZZ-HLCs. We found evidence of gene network clusters indicating inflammatory (*e.g. IL18*) and cellular stress responses, (*e.g.* calnexin (*CANX*), calreticulin (*CALR*), BiP/GRP78 (*HSPA5*), ERdj5 (*DNAJC10*), ERdj6 (*DNAJC3*), *DNAJB11*, GRP94 (*HSP90B1*) and GRP170 (*HYOU1*) (Fig. S2). The differential gene expression profile of ZZ-HLCs not only appeared to mirror components of the A1AT-deficient pathology that have previously been identified (*e.g. CANX* and *CALR*), but also uncovered new genetic networks. Of particular interest were those genes linking misfolding-induced inflammatory responses to mitochondrial abnormalities, since our electron microscopy studies demonstrated ZZ-HLC populations contained markedly abnormal mitochondrial phenotypes (Fig. 6D), as also previously described in human tissue and HLCs.^31^ We sought to validate the importance of these genes by examining differential gene expression at the nexus of inflammatory (*IL-18* and *CASP4*) and unfolded protein (*CANX* and *CALR*) clusters in a second hiPSC cell model of A1ATD derived from a different patient in a different laboratory.^32^ All four genes were also found to be upregulated in PiZZ *vs.* PiMM cells, suggesting their importance for future investigations (Fig. S3). Taken together, these data demonstrated that the biomarkers and putative new mechanisms identified by our approach could be clinically relevant for a broad number of A1AT-deficient patients.

## Discussion

Despite the classification of A1ATD as a monogenetic disease, the underlying molecular mechanisms causing liver pathology are poorly understood. hiPSCs offer an appealing model to interrogate such questions, but have until now been hampered by concerns over whether *in vitro* signatures reflect the human disease. To address this, our study characterised the molecular and cellular signatures of ZZ-HLCs in comparison to human tissue. We first determined that, as in patients, only a subset (∼40%) of ZZ-HLCs contained A1AT polymers and that these polymers are contained within the ER but physically isolated from the rest of the ER lumen. Of note, Tafaleng *et al.* previously utilised ZZ-HLCs and ZZ liver samples to establish the presence of globular inclusions and dilated ER.^12^ Our studies elaborate on these observations by using ZZ/RR-HLCs to provide real-time proof of physical ER fractionation and absent functional connection between abnormal ER structures. Importantly, our analyses of liver samples from patients with A1ATD also suggest that the disease could be regionalised as these globular inclusions were mainly located in the lobule periphery. Thus, disease progression mechanisms could be associated with liver zonation. To further shed light on the molecular mechanisms governing this specific type of intracellular A1AT retention, we then compared genome-wide RNA-seq and ER-specific proteomic datasets of ZZ-HLCs *vs.* RR-HLCs. The foremost dysregulated pathways, both on a transcriptomic and proteomic level, were associated with known pathways of the misfolded protein response. In addition, our analyses also revealed novel molecular proteins and pathways important in chaperone function, inflammation, mitochondrial dysfunction and malignancy, which we validated in human tissue.

Despite the persistent molecular and functional similarities of HLCs with foetal hepatocytes,^16^ this work has shown the adequacy of ZZ-HLCs to replicate the molecular pathways of PiZZ hepatocytes. Their developmental immaturity offers the opportunity to study the emergence, early onset and progression of A1ATD, even at stages in which the disease is still asymptomatic in most patients.^33^ A good example of this is the photobleaching assays (Fig. 3, Fig. 4), which clearly demonstrated that ZZ-HLCs have significant loss of protein mobility, ER luminal communication and in some cases complete disruption of ER cisternae into isolated units. Whilst it has previously been demonstrated that even small deviations in protein diffusion rates can lead to biologically relevant changes,^34^ our observations offer a novel insight into the subcellular anatomy of how this occurs in A1ATD. Such understanding is likely to be of clinical significance since cells harbouring fragmented ER inclusions have been shown to be more prone to apoptotic cell death and rely on replacement by globule-devoid hepatocytes with enhanced cell division rates.[35], [36] However, whether A1AT polymer sequestration into globules is a protective cellular response or signals a state of injury in this setting (or even both)[36], [37] is currently not known. Further *in vitro* studies, combined with human histological specimens and clinical data may allow us to deconstruct this problem by examining whether there is a link between the severity and number of ER inclusions, and an association between the severity and progression of liver disease. In a similarly important and clinically relevant manner, early identification of A1AT-deficient patients who are likely to progress to cancerous states represents an unmet but urgently required clinical challenge. Whilst AKR1B10 has been associated with hepatocellular carcinoma,[38], [39] its co-localisation within cells containing polymers (Fig. 6C) suggests an association of this protein with A1ATD. The presence of AKR1B10 may therefore be a direct consequence of polymerised protein accumulation, characterising a subset of hepatocytes primed towards malignancy, making AKR1B10 a potential tissue biomarker for hepatocellular carcinoma risk stratification. Other proteins with known liver/cancer associations such as cysteine cathepsin family member CTSO[40], [41] were also identified in our ZZ-HLC cells and may similarly provide prognostic and mechanistic insights into why individuals with A1ATD are at increased risk of tumourigenicity.^42^

Understanding molecular pathways linking the polymerisation of Z A1AT to hepatic disease is central to the design of future therapeutics. Our genome and proteome analyses (Fig. 5) identified differentially regulated genes known to have an association with A1ATD (*e.g.* proteins of the ER-associated degradation machinery) as well as novel factors required to recognise and bind mutant glycoproteins, retro-translocate them from the ER lumen to the cytosol and tag them for proteasomal degradation. With the reassurance provided by this biological baseline, we also identified molecules that could offer insights into the pathophysiology of disease using transcriptomics and proteomics. The twenty highest-expressed, differentially upregulated genes in ZZ-HLCs were all of mitochondrial origin and associated with tasks of meeting higher energy demands. This manifested as physical disruption of the mitochondria (Fig. 6D) which although described previously,^43^ is not yet comprehensively characterised with respect to its key molecular participants.^31^ Our data provide new insight into the link between ER misfolding and mitochondrial disruption. Of particular interest, ER-stress response proteins ARMET (*MANF*),^44^ OASIS (*CREB3L1*),^45^ quality control chaperone proteins (*HSP90*,^46^ Calreticulin,^47^ Calnexin^48^) and pro-inflammatory chemokines, cytokines and receptors (“inflammasome”) known to mediate cytotoxic stress and inflammation (IL-1, IL-18, Caspase-4, TNF),[49], [50] were upregulated in ZZ-HLCs. Interestingly, of the five most highly upregulated genes in ZZ-HLCs reported by Wilson *et al.*, *CASP4* and *CFH* also appeared in our list of 1,675 significant genes differentially regulated between ZZ-HLCs and RR-HLCs (FDR < 5%).^32^ We therefore propose these targets, known also to mediate inter-organelle communication, as potentially being pivotal to disease and worthy of further research. Finally, this work also indicates that a more direct drug screening approach to target these novel pathways in A1ATD is merited.

## Financial support

CPS is funded through a Children’s Liver Disease Foundation (CLDF) studentship. DAL is funded by the Medical Research Council, Wellcome Trust, GlaxoSmithKline, the Rosetrees Trust, EPSRC and UCLH NIHR Biomedical Research Centre. LV, PM, MCB, NH are funded by ERC Relieve-IMDs and ERC advanced grant New-Chol, Cambridge University Hospitals National Institute for Health Research Biomedical Research Centre, and the core support grant from the Wellcome Trust and Medical Research Council to the Wellcome Trust – Medical Research Council Cambridge Stem Cell Institute. STR is funded by an MRC Clinician Scientist Fellowship award.

## Conflict of interest

STR and LV are scientific founders of Definigen Ltd. (shares and consultancy). DAL is working with GlaxoSmithKline to develop small molecules that block the intracellular polymerisation of α_1_-antitrypsin. All other authors declare no conflicts of interest.

Please refer to the accompanying ICMJE disclosure forms for further details.

## Authors’ contributions

STR, DAL and LV conceived the project. CPS led the experimental procedures with support from all other co-authors. CPS, STR, DAL and LV analysed the data and wrote the manuscript.
