# Supplementary material for: hiPSC hepatocyte model demonstrates the role of unfolded protein response and inflammatory networks in α1-antitrypsin deficiency
Source: J Hepatol. 2018 Oct;69(4):851–60. doi: 10.1016/j.jhep.2018.05.028 (PMC6562205; doi:10.1016/j.jhep.2018.05.028)
Supplement: Supplementary data 3 [file CTAT_table.pdf]

EASL | JOURNAL OF HEPATOLOGY

Journal of Hepatology

CTAT methods

Tables for a "Complete, Transparent, Accurate and Timely account" (CTAT) are now mandatory for all revised submissions. The aim is to enhance the reproducibility of methods.

- Only include the parts relevant to your study
- Refer to the CTAT in the main text as 'Supplementary CTAT Table'
- Do not add subheadings
- Add as many rows as needed to include all information
- Only include one item per row

If the CTAT form is not relevant to your study, please outline the reasons why:

Detailed methods are also listed in the Supplementary Materials.

1.1 Antibodies

Name	Supplier	Cat no.
Rb pAb anti human A1AT	Sigma	A0409
Rb pAb anti human ALB	R&D Systems	MAB1455
Rb pAb anti human HNF4α	Santa Cruz	sc-8987 H-171
Rb mAb anti human AKR1B10	Abcam	ab192865

1.2 Cell lines

Name	Citation	Supplier	Cat no.	Passage no.	Authentication test method
hiPSCs derived in-lab (Rashid et al., JCI, 2010).					

1.3 Organisms

Name	Citation	Supplier	Strain	Sex	Age	Overall n number
Not applicable						

1.4 Sequence based reagents

Name	Forward primer sequence	Reverse primer sequence
Ubiquitin C	ATTTGGGTCGCGGTTCTTG	TGCCTTGACATTCTCGATGGT
Porphobilinogen deaminase	GGAGCCATGTCTGGTAACGG	CCACGCGAATCACTCTCATCT
Ribosomal protein, large, P0	GGCGTCCTCGTGGAAGTGAC	GCCTTGCGCATCATGGTGTT
α 1-antitrypsin	AGACCCTTTGAAGTCAAGCGACC	CCATTGCTGAAGACCTTAGTGATGC
α -fetoprotein	AGAACCTGTCACAAGCTGTG	TGGTAGCCAGGTCAGCTAAA
Albumin	CCTTTGGCACAATGAAGTGGGTAACC	GACAGCAAGCTGAGGATGTC
Cytochrome P450, 3A4	TGTGCCTGAGAACACCAGAG	GTGGTGGAAATAGTCCCGTG
Cytochrome P450, 3A7	GAAACACAGATCCCCCTGAA	TCAGGCTCCACTTACGGTCT
Hepatocyte nuclear factor 4 α	CATGGCCAAGATTGACAACCT	TTCCCATATGTTCCTGCATCAG
Calreticulin	GGCACTTGGATCCACCCAGA	CTGCTGCCTTTGTTACGCCC
Calnexin	GTCCCCGGGAGGCTAGAGATCA	AGGAGGAGCAGTGGTATCTGGT
Caspase 4	ACAGAGGCTGTTCCCTATGGC	AGCCTCCATATTCGGATGAGCTTT
Interleukin 18	AGCTGAAGATGATGAAAACCTGGA	GCCATACCTCTAGGCTGGCT

1.5 Biological samples

Description	Source
Human liver biopsy sections	King's College London Liver Institute

1.6 Deposited data

Name of repository	Identifier	Link
RNA-seq data	accession number E-MTAB-6781.	www.ebi.ac.uk/arrayexpress

1.7 Software

Software name	Manufacturer	Version
Not applicable.		

1.8 Other (e.g. drugs, proteins, vectors etc.)

Not applicable.

1.9 Please provide the details of the corresponding methods author for the manuscript:

Prof. Ludovic Vallier: lv225@cam.ac.uk

2.0 Please confirm for randomised controlled trials all versions of the clinical protocol are included in the submission. These will be published online as supplementary information.

Not applicable.
