# Supplementary material for: hiPSC hepatocyte model demonstrates the role of unfolded protein response and inflammatory networks in α1-antitrypsin deficiency
Source: J Hepatol. 2018 Oct;69(4):851–60. doi: 10.1016/j.jhep.2018.05.028 (PMC6562205; doi:10.1016/j.jhep.2018.05.028)
Supplement: Supplementary data 1 [file mmc1.pdf]

hiPSC hepatocyte model demonstrates the role of unfolded protein response and inflammatory networks in α_1-antitrypsin deficiency

Charis-Patricia Segeritz, Sheikh Tamir Rashid, Miguel Cardoso de Brito, Maria Serra Paola, Adriana Ordonez, Carola Maria Morell, Joseph E. Kaserman, Pedro Madrigal, Nicholas Hannan, Laurent Gatto, Lu Tan, Andrew A. Wilson, Kathryn Lilley, Stefan J. Marciniak, Bibekbrata Gooptu, David A. Lomas, Ludovic Vallier

Table of contents

Supplementary materials and methods……………………………………………2

Table S1……………………………………………………………………………3

Fig. S1……………………………………………………………………………12

Fig. S2……………………………………………………………………………12

Fig. S3……………………………………………………………………………13

Supplementary materials and methods

RNA extraction and gene expression analysis with quantitative PCR

Cells in a 12-well plate were washed once with PBS (Gibco, Cat. No. 14190-94) and then lysed with 350 µL/well of RNA lysis buffer provided in the RNA extraction kit (Sigma, Cat. No. RTN350-1KT). The lysate was transferred to an Eppendorf tube and frozen at -80 °C until RNA extraction. The protocol for RNA extraction followed the manufacturer's manual. RNA concentrations were measured on the Nanodrop (Thermo Scientific). To convert total RNA into cDNA for quantitative PCR (qPCR), each RNA sample was diluted in nuclease free water (Ambion, Cat. No. AM9937) to a concentration of 500 ng in 11.875 µL. A volume of 0.5 µL random primers (Promega, Cat. No. C1181) and 1 µL dNTPs (Promega, Cat. No. U1511) were added to each RNA sample. The mix was incubated at 65 °C for 5 min and then snap-cooled on ice. Then the following master mix (6.625 µL) was prepared and added to each RNA sample: 5x 1st strand buffer (Invitrogen, Cat. No. 18064-071): 4 µL; 0.1 M Dithiothreitol (DDT) (Invitrogen, Cat. No. 18064-071): 2 µL; RNaseOUT (Invitrogen, Cat. No. 10777-019): 0.5 µL; SuperScript II (Invitrogen, Cat. No. 18064-071): 0.125µL. The final reaction volume of 20 µL was incubated for the following times: 25 °C x 10 min, 42 °C x 50 min, 70 °C x 15 min. The generated cDNA samples were stored at 4 °C or frozen at -20 °C until processed. qPCR reagents and cDNA samples were thawed on ice and 20 µL of cDNA was diluted with 580 µL nuclease-free water (Cat. No. Ambion, AM9937) to a total volume of 600 µL. The following total reaction volume of 15 µL was generated per well: Forward primer (5µM) (Sigma): 0.6 µL; reverse primer (5µM) (Sigma): 0.6 µL; 2x Sensi Mix SYBR Lo-ROX (Bioline, Cat. No. QT625-20): 7.5 µL; nuclease-free H_2O (Ambion, Cat. No. AM9937): 1.3 µL, cDNA template: 5 µL. List of primers sequence is provided in supplementary table 1. The reactions were performed using a Stratagene Mx3005P thermal cycler (Agilent Technologies). The cDNA template was first denatured at 94 °C for 5 min, then cycled at 94 °C for 30 s, 60 °C for 30 s (annealing of the primers), and 72 °C for 30 s, followed by final extension at 72 °C for 10 min after

completion of 40 cycles. In order to quantify relative gene expression (ΔCt method), qPCR reactions for control "housekeeping" genes were performed in the same run as assayed genes and used to normalise for total cDNA concentrations (e.g. ubiquitin C; Silver et al., 2008). Statistical analysis by unpaired, parametric t-test: significance $p \leq 0.05$ (*), $p \leq 0.01$ (**), $p \leq 0.001$ (***) and $p \leq 0.0001$ (****).

Table S1. qPCR primer sequences list.

Gene name / abbreviation		Forward primer sequence	Reverse primer sequence
Ubiquitin C	UBC	ATTTGGGTCGCGGTTCTTG	TGCCTTGACATTCTCGATGGT
Porphobilinogen deaminase	PBGD	GGAGCCATGTCTGGTAACGG	CCACGCGAATCACTCTCATCT
Ribosomal protein, large, P0	RPLP0	GGCGTCCTCGTGGAAGTGAC	GCCTTGCGCATCATGGTGTT
α 1-antitrypsin	A1AT	AGACCCTTTGAAGTCAAGCGACC	CCATTGCTGAAGACCTTAGTGATGC
α -fetoprotein	AFP	AGAACCTGTCACAAGCTGTG	TGGTAGCCAGGTCAGCTAAA
Albumin	ALB	CCTTTGGCACAATGAAGTGGGTAACC	GACAGCAAGCTGAGGATGTC
Cytochrome P450, 3A4	CYP3A4	TGTGCCTGAGAACACCAGAG	GTGGTGGAAATAGTCCCGTG
Cytochrome P450, 3A7	CYP3A7	GAAACACAGATCCCCCTGAA	TCAGGCTCCACTTACGGTCT
Hepatocyte nuclear factor 4 α	HNF4 α	CATGGCCAAGATTGACAACCT	TTCCCATATGTTCCTGCATCAG
Calreticulin	CALR	GGCACTTGGATCCACCCAGA	CTGCTGCCTTTGTTACGCCC
Calnexin	CANX	GTCCCCGGGAGGCTAGAGATCA	AGGAGGAGCAGTGGTATCTGGT
Caspase 4	CASP4	ACAGAGGCTGTTCCCTATGGC	AGCCTCCATATTCGGATGAGCTTT
Interleukin 18	IL-18	AGCTGAAGATGATGAAAACCTGGA	GCCATACCTCTAGGCTGGCT

Immuncytochemistry and Immunofluorescence Staining

Cells were fixed for 20 min at 4° C in 4 % PFA (Alfa Aesar, Cat. No. 43368) and then washed three times in PBS (Gibco, Cat. No. 14190-94). Cells were blocked and permeabilised with PBS containing 10 % donkey serum (Bio-Rad, Cat. No. C06SB) and 0.1 % Triton-X (Sigma, Cat. No. T8787-250ML) for 20 min at 20 °C. Cells were subsequently incubated overnight at 4 °C with primary antibody diluted in 1 % donkey serum in PBS as follows: Rb pAb anti human A1AT used at 1:100 (Sigma, Cat. No. A0409); Rb pAb anti human ALB used at 1:100 (R&D, Cat. No. MAB1455); Rb pAb anti human HNF4α used at 1:100 (Santa Cruz, Cat. No. sc-8987 H-171). Cells were then washed three times in PBS and incubated with anti-mouse or anti-rabbit

secondary antibodies for 2 h at 20 °C. Unbound secondary antibody was removed by three washes in PBS. Hoechst 33258 was added to the first wash at 1:10,000 to visualise the nucleus (Sigma-Aldrich, Cat. No. H3569).

For AKR1B10 immunofluorescent staining on paraffin-embedded tissue blocks, 5μm slides from 18 patients were dewaxed and boiled with TRIS EDTA PH9 (AbCam ab93684). After the blocking, slides were incubated with AKR1B10 antibody at 1:100 (AbCam ab192865), followed by alkaline phosphatase (AP)-conjugated secondary antibody and the avidin/biotin AP system (Vectastain ABC Kit; Vector Laboratories, Burlingame, CA). VECTOR Red Alkaline Phosphatase (Red AP) Substrate was applied and slides were counterstained with Hematoxylin and scanned with the Hamamatsu NanoZoomer.

P450-Glo™ CYP3A4.

Using the P450-Glo™ CYP3A4 Assay (Promega, Cat. No. V9002), cells were assayed in triplicate wells and according to the manufacturer's instructions. Cytochrome activity levels were captured using a P450-GloMax 96 microplate luminometer (Promega), measured as relative luminescence unit (RLU), and normalised to the assay volume and per $1x10^6$ cells. The cell number was determined by dissociating cells of the assayed wells with TrypLE™ (Life Technologies, Cat. No. 12563-029), counting the cells in a 0.4 % Trypan Blue solution on a haemocytometer. Statistical analysis by unpaired, parametric t-test: significance $p \leq 0.05$ (*), $p \leq 0.01$ (**), $p \leq 0.001$ (***) and $p \leq 0.0001$ (****).

Serum protein secretion assays

To determine the concentrations of secreted albumin and A1AT, cell culture media were collected after 48 h of incubation with hiPSC-derived hepatocytes differentiated to day 35. Cell supernatants were analysed in triplicates using an in-house 2-site

microtire plate-based time-resolved fluorescence immunoassays (DELFIA). For the anti-albumin assay, the following antibodies were used: Capture Antibody: Polyclonal Goat Anti-Human Albumin antibody (Bethyl Laboratories), Detection Antibody: Polyclonal Rabbit Anti-Human Albumin antibody (Dako), Europium-Labelled Reagent: Europium-Labelled Anti-Rabbit IgG (Perkin Elmer). For the anti-A1AT assay, the following antibodies were used: Capture Antibody: Polyclonal Rabbit Anti-Human AAT antibody (Siemens Healthcare), Detection Antibody: Biotinylated Polyclonal Goat Anti-Human AAT antibody (AbCam), Europium-Labelled Reagent: Europium-Labelled Streptavidin (Perkin Elmer). The albumin assay was calibrated to a human serum solution (BioRad Maltiqual Level 1) and diluted to prepare a 8-point standard curve with concentrations ranging from 0 to 1000 ng/mL. The A1AT assay was calibrated using BNII Protein Calibrator (Siemens Healthcare) and diluted to prepare a 9-point standard curve with concentrations ranging from 0 to 500 ng/mL. Microtitre plates, assay buffer and enhancement solutions for both assays were all supplied by Perkin Elmer. Statistical analysis by unpaired, parametric t-test: significance $p \leq 0.05$ (*), $p \leq 0.01$ (**), $p \leq 0.001$ (***) and $p \leq 0.0001$ (****).

α1-antitrypsin pulse-chase radiolabelling

We employed pulse-chase radiolabelling to determine the nature and kinetics of intracellular A1AT, its post-translational processing and secretion in ZZ-HLCs and RR-HLCs [9]. To that end, hiPSCs were hepatically differentiated to day 35 and then starved in methionine (Met)- and cysteine (Cys)-free pulse medium for 90 min. Subsequent incubation of the starved cells with 1.3 MBq of ^{35}S-Met/Cys for 30 min at 37 °C resulted in incorporation of radioactive amino acids. Cells were then washed and incubated in L-Met and L-Cys-supplemented chase medium for 1 h, 2 h and 4 h before collection of cell supernatants and harvesting of the cells. The total protein lysate was extracted from cells using Nonidet P-40 lysis buffer (150 mM NaCl, 50 mM Tris-HCl pH

7.5, 1 % Nonidet P-40), containing a 25 mM protease inhibitor mixture (Roche, Cat. No. 04693132001), followed by centrifugation at 2400 x g. Immunoprecipitation of monomeric and polymeric A1AT from lysates and supernatants was achieved with the polyclonal anti-human A1AT antibody and monoclonal 2C1 anti-A1AT polymer antibody generated in the group of Prof. D. A. Lomas [30]. The immunoprecipitated and radiolabelled proteins were then resolved on a 10 % v/v SDS-PAGE gel and subsequently visualised and quantified on a Cyclone Phosphor Imager (Packard Instrument Co.).

Electron microscopy

hiPSC-derived hepatocytes differentiated to day 35 were digested with TrypLE^{TM} (Life Technologies, Cat. No. 12563-029) and centrifuged for 5 min at 100 x g. The cell pellet was washed with PBS, and fixed with 2.5 % v/v glutaraldehyde solution overnight at 4 °C. Post-fixation was carried out with 0.2 M cacodylate and 2 % w/v osmium tetroxide for 1.5 h. After dehydration, the cells were rinsed with propylene oxide before being infiltrated with epoxy resin. After a 16 h polymerisation step at 60 °C, the Thermanox coverslips were peeled away leaving the cell monolayer inside the resin block. Sixty nanometer sections were cut and stained using an electron microscopy stain (Leica Microsystems) and then imaged at 100 kV in a Hitachi, H-7650 transmission electron microscope.

Subcellular fractionation and preparation of ER-enriched fractions for mass spectrometry

Stock solutions for subcellular fractionation. Solution A: OptiPrep (60 % iodixanol in water, Sigma, Cat. No. D1556-250ML); Solution B (washing buffer): 0.25 M sucrose, 1 mM EDTA, 20 mM HEPES (pH 7.4); Solution C: 0.25 M sucrose, 6 mM EDTA, 20 mM HEPES (pH 7.4); Solution D: 5 volumes Solution A, 1 volume Solution C; Gradient for 10 % OptiPrep: 2 mL Solution D, 8 mL Solution B; Gradient for 30 % OptiPrep: 6 mL

Solution D, 4 mL Solution B. Stock solutions prepared ahead of the experiment were stored at 4 °C.

ZZ-HLCs and RR-HLCs grown in three p100 plates each were placed on ice, washed once with ice cold PBS, once with solution B and were then mechanically harvested using a cell scraper into a final volume of < 1 mL. This solution was aspirated into a 1 mL syringe and introduced into a ball-bearing EMBL cell-homogeniser (Isobiotec, Heidelberg, Germany) containing 0.6 mL Solution B and a 8.010 mm-sized ball. Cells were then mechanically disrupted by 30 passages through the homogeniser embedded in ice. Disruption was confirmed by light microscopy of a small subsample. The cell suspension was centrifuged at 800 x g for 10 min at 4 °C on a desktop centrifuge to pellet the nuclei. Next, density gradient centrifugation was used to isolate the ER-enriched fractions through a continuous iodixanol gradient (OptiPrep, Axis-Shield, Oslo, Norway). To that end, the supernatant containing the subcellular membranes was diluted to a final concentration of 35 % OptiPrep by adding 1.4 mL solution D to 600 µL supernatant. This 2 mL solution was then pipetted into the bottom of an Ultra-Clear™ centrifugation tube (Beckman Coulter, Cat. No. 344057) and with a Densi-Flow Density Gradient Fractionator (Labconco, Kansas City) carefully layered on top by 30 % and 10 % OptiPrep solutions. Each tube was carefully balanced with Solution B and then centrifuged at 200,000 x g for 2 h at 4 °C using an Sw55Ti rotor (Beckman Coulter). The different microsomal fractions in the centrifuged tube were captured by taking off sequential 500 µL fractions and storing them at -20 °C.

<u>Preparation of ER-enriched fractions for mass spectrometry</u>

The subcellular ZZ-HLC and RR-HLC fractions (See supplementary material) were investigated for ER enrichment by Western blot (See supplementary material) against the ER-resident protein KDEL. KDEL-positive fractions were pooled and concentrated to < 200 µL and resolved on a 1D SDS-PAGE gel stained with Coomassie Blue. Each lane was cut into ten gel pieces, destained, treated with dithiothreitol to reduce

disulphide bonds and free cysteine moieties were alkylated with iodoacetamide. The gel pieces were then washed, dehydrated and tryptically digested overnight at 37 °C with 20 ng/µL tryptic solution (Promega, Cat. No. V5111) in 50 mM ammonium bicarbonate. After digestion, the supernatant was pipetted into a sample vial and loaded onto an autosampler for automated LC-MS/MS analysis.

Mass spectrometry

Three biological replicates of RR-HLCs and ZZ-HLCs were analyzed in six label-free mass spectrometry acquisitions. All LC-MS/MS experiments were performed using a nanoAcquity UPLC (Waters Corp., Milford, MA) system and a LTQ Orbitrap Velos hybrid ion trap mass spectrometer (Thermo Scientific, Waltham, MA).

Post-run, all MS/MS data were converted to mgf files and these were submitted to the Mascot search algorithm (Matrix Science, London UK) and searched against the Uniprot human database, using a fixed modification of carbamidomethyl (C) and a variable modification of oxidation (M) with a peptide tolerance of 20 ppm (MS) and 0.1 Da (MS/MS). Peptide identifications were accepted if they could be established at greater than 95.0 % probability. For further analysis and quantification, peptides were subsequently processed with MaxQuant v1.0. Peptide counts were used for the label-free quantitation (LFQ) and a maximum posterior error probability (PEP) of 0.1 was applied.

Data filtering: All samples that achieved zero values for any of the assayed proteins (“missing values”) were set to “NA”, while proteins were removed that did not have any quantitation data matched to any of the six samples. Furthermore, known protein contaminants (CON) and proteins stemming from the reverse data base (REV) that did not share peptides with genuine majority protein IDs were removed.

Data normalisation: Prior to statistical analyses, all six samples were scaled towards an experiment-wide median value.

Western blotting

15 µL cell lysate of each fraction was mixed with 5 µL loading buffer (4x) (Invitrogen, Cat. No. NP0007) and 0.2 µL β-Mercaptoethanol (4 %) (Sigma, Cat. No. M3148-2ML) and then denatured for 5 min at 90 °C. The sample mix was loaded onto a 4-12 % Bis-Tris Gel (Invitrogen, Cat. No. NP0321BOX) alongside a protein ladder (Bio-Rad, Cat. No. 161-0374) and run for 45 min at 200 V. Proteins were transferred to a PVDF membrane (Bio-Rad, Cat. No. 162-0177) in transfer buffer (Novex, Cat. No. NP0006-1) for 1 h at 100 V and then prepared for blotting by incubating the membrane for 30-60 min under agitation in blocking solution (5 % milk in PBS and 0.05 % Tween-20 (Sigma, Cat. No. P9416-100ML)). The membrane was then incubated in blocking solution containing the primary antibody (mouse monoclonal anti-KDEL (10C3) used at 1:250 (Enzo Life Sciences, Cat. No. ADISPA-827)), rocking it for 1 h at 20 °C or at 4 °C overnight. After washing the membrane three times in PBS, each for 5 min, the membrane was stained as above with the secondary antibody (anti-mouse IgG-peroxidase produced in goat (Sigma, Cat. No. A0545-1ML or A2554-1ML)), diluted at 1:10,000 in blocking solution. After thoroughly washing the membrane (three times for 5 min each), it was covered with detection solution (GE Healthcare, Cat. No. RPN2232) and placed into a cassette. In a dark room, the protein blot was exposed to film and inserted into the developer.

Photobleaching analysis in hIPSCs derived hepatocytes

Per well of a 6-well plate containing HLCs differentiated to day 35, 4 µg of the respective plasmid DNA (a luminal ER-GFP plasmid or a fluorescent ER membrane plasmid which was composed of a GFP tag added to the cytoplasmic end of an ER signal-anchor membrane protein) was diluted in 0.25 mL of Opti-MEM medium (Gibco, Cat. No. 51985-026) [35] [20] 10 µL of Lipofectamine 2000 (Invitrogen, Cat. No. 11668019) was diluted in 0.25 mL of Opti-MEM medium. The diluted DNA and Lipofectamine were mixed and incubated for 20 min. Culture medium was aspirated

from the wells and replaced with 1 mL/well of Opti-MEM. Drop-by-drop, 0.5 mL of the DNA/Lipofectamine mix was added to each well and the plate was slightly shaken before placing it in the incubator for 24-48 h of transfection. Transfection efficiencies were typically between 5-10%. Fluorescence recovery after photobleaching and fluorescence loss in photobleaching experiments (FRAP/FLIP) were carried out by imaging transiently transfected, live cells on a 37 °C environmental controlled stage of a confocal microscope system (DuoScan, Carl Zeiss Inc.) with a 63x/1.4NA oil objective. Full laser power was employed for photobleaching of small regions of interest and fluorescence recovery or loss was monitored over 0.2-second time intervals. Fluorescence loss curves were obtained by transforming fluorescence intensities into a percentage scale in which the pre-bleach time point represented 100% of fluorescence intensity.

RNA-seq data analysis

Quality control. The quality of bases called was assessed using the Sickle v1.210. Reads containing any Ns (parameter -n) or those which did not pass the sliding window with quality and length thresholds -q 20, -l 30 were discarded. Less than 3.2% of all reads were removed in this step.

Read alignment. Reads kept were mapped to Ensembl GRCh37 (release 75, February 2014) of the Homo sapiens genome using TopHat 2.0.10 (D. Kim et al., 2013) and Bowtie 2.1.0 (Langmead, Trapnell, Pop, & Salzberg, 2009). We supplied TopHat with the gene model annotations and known transcripts using the option '–GTF' (GRCh37.75). Thus, only reads which did not map to the transcriptome were then mapped onto the genome. Only read alignments with mapping quality score MAPQ > 10 were kept for further processing.

Reproducibility of biological replicates. The software tool featureCounts (Liao, Smyth, & Shi, 2014) was used to calculate the number of reads which map uniquely to exons in Ensembl GRCh37.75, and then associated those to the meta-feature

'gene_id' ('-t exon -g gene_id'). We computed rank-based Spearman correlation coefficient ρ between read counts across biological replicates and found that biological replicates were reproducible for each genotype at approximately ρ = 0.9 when using read counts (as calculated by featureCounts) for 63677 gene features. In addition, calculations of the Pearson Correlation co-efficient (r) using FPKM values for RNAs that were detected in both cell lines (FPKM > 0.0) resulted in values r > 0.94 for all pair-wise comparisons. Our principal component analyses (PCA) confirmed highly reproducibility of the given datasets associated with ZZ- and RR-HLC triplicates.

Validation of the *SERPINA1* point mutation. The selective gene-editing of A1AT-ZZ hiPSCs to restore the wild-type A1AT genotype was confirmed by determining the identity and the number of bases called at the coordinate chr14:94844947, the specific site of the Z mutation (Glu342Lys, rs28929474). These in-depth analyses demonstrate the successful application of molecular gene-editing techniques to correct the SERPINA1 point mutation and showed the generation of two distinct SERPINA1-specific genomes in ZZ- and RR-HLCs.

Differential gene expression analysis. Differential gene expression analyses were performed using the R/Bioconductor package DESeq2 v1.4.5 (Love et al., 2014) under default parameters to test for the effect of the corrected point mutation. 1675 genes showed differential expression for a FDR < 5 % (Benjamini-Hochberg FDR adjusted p-value < 0.05, Wald tests) when compared between the conditions ZZ-HLCs and RR-HLCs.

Gene expression level-based clusters. FPKM values were computed using the function 'fpkm' in the Bioconductor Package DESeq2. Length of the exons for each gene was extracted form Ensembl (release 75). No scaling was applied to the log2 (FPKMs). Hierarchical clustering was by performed by 'hclust' and 'dist', which were used by default in heatmap.2 function in R.

<u>SUPPLEMENTARY FIGURES AND LEGENDS:</u>

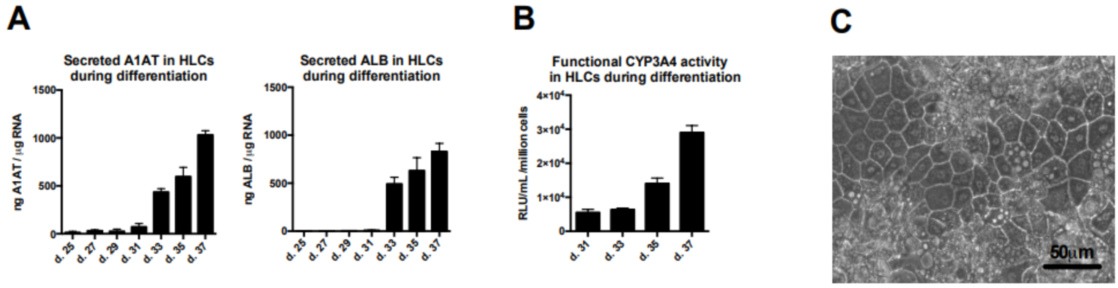

<u>Fig. S1.</u> HLCs differentiated for a prolonged period of time increase expression of hepatic markers. (A) DELFIA of secreted α_1-antitrypsin and albumin of HLC differentiated for 37 days. n=12. (B) CYP-P450 GLO functionality of HLCs differentiated for 37 days. n=12. (C) Bright-field image of a RR-HLC culture differentiated to day 35.

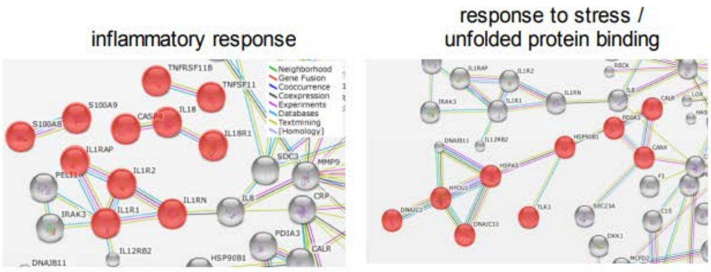

<u>Fig. S2</u>. STRING Analysis was performed with the highest confidence view (score = 0.900) on the 959 genes upregulated in ZZ-HLCs. Each node represents a connected protein. Disconnected proteins are not shown.

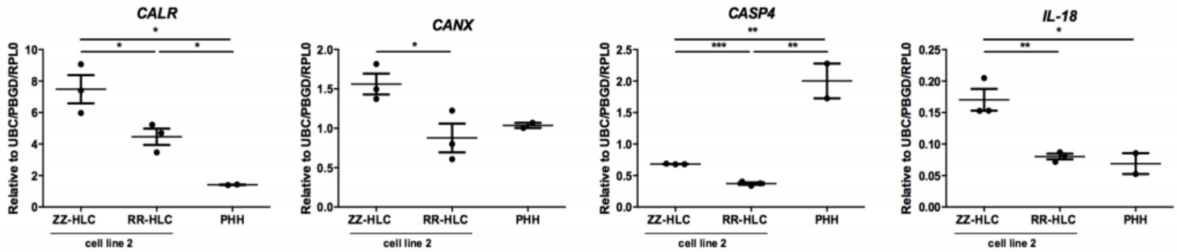

Fig. S3. Q-PCR analyses confirming that markers of the unfolded protein response and inflammatory gene network are also upregulated in ZZ-HLCs generated from different hIPSC lines. PHH (primary human hepatocytes).
